# Validity of assessing level walking with the 2D motion analysis software TEMPLO and reliability of 3D marker application

**DOI:** 10.1038/s41598-024-52053-z

**Published:** 2024-01-16

**Authors:** Klaus Widhalm, Sebastian Durstberger, Andrea Greisberger, Brigitte Wolf, Peter Putz

**Affiliations:** FH Campus Wien, University of Applied Sciences, Health Sciences, Vienna, Austria

**Keywords:** Rehabilitation, Orthopaedics, Osteoarthritis

## Abstract

In gait analysis, knowledge on validity and reliability of instruments and influences caused by the examiner’s performance is of crucial interest. These measurement properties are not yet known for commonly used, low-cost two-dimensional (2D) video-based systems. The purpose of this study was to assess the concurrent validity of a video-based 2D system against a three-dimensional (3D) reference standard, as well as the inter-rater reliability, and test–retest reliability of 3D marker application. Level walking was captured simultaneously by a 2D and a 3D system. Reflective markers were applied independently by three raters and repeated by one rater on a second day. We assessed the agreement between the two systems, as well as reproducibility, and inter-rater agreement of derived spatio-temporal parameters and sagittal kinematics. Nineteen healthy participants completed this study. 2D gait analysis provides a possibility to accurately assess parameters such as stride time, stride length, gait velocity, and knee RoM. Interrater and test–retest reliability of 3D gait analysis are generally acceptable, except for the parameters toe-off and pelvic RoM. This is the first study to publish measurement properties of a commercially available 2D video-based gait analysis system, which can support interpretation of gait pattern near the sagittal plane.

## Introduction

The quality and accuracy of instrumented gait analysis in level walking depends on the nature of gait dysfunction and the measurement technology used^1–5^. The influence of rater performance on the reliability of outcomes has been assessed for highly instrumented methods like 3D optoelectronic systems^1^, as well as for observational ratings^6^. Inter-rater reliability of gait parameters was shown to be an important metric in a team of raters performing gait analysis^7^. Test–retest reliability is considered a quality benchmark in the analysis of longitudinal changes in level walking^8^. However, compared to the number of studies on level walking, few papers have been published on inter-rater and test–retest reliability, which also serves as a standardization basis for the respective gait labs.

To optimize observational gait analysis, some institutions have implemented standardized instrumental three-dimensional gait analysis (3DGA) by means of optoelectronic movement analysis systems to assess level walking. 3DGA is a widely accepted reference standard for assessing gait parameters if applied by a rater following marker placement training^9^. However, for reasons of cost and space, 3DGA is not yet very common and cannot easily be used in extramural settings. For assessing spatio-temporal parameters, pressure distribution platforms^10,11^, LED bars, and inductive walkway systems^12^ are available and were tested for reliability with partly excellent results. To obtain additional 3D kinematic parameters, systems based on inertial measurement units were introduced^13^. Low-cost and mobile depth-finding camera-equipped game consoles may not accurately obtain lower body kinematic data, but show potential for spatio-temporal assessments^14,15^. Overall, it was concluded that validation studies on some of these technologies are of limited quality, but reliability was better investigated than concurrent validity, and spatio-temporal gait parameters consistently outperformed planar joint angle data^16^. To our knowledge, only four commercially available video-based movement analysis systems have been assessed for their accuracy in level walking^5^.

Considering the advantages of two-dimensional (2D) video-based kinematic analyses with marker tracking, which are inexpensive, commonly available, and highly mobile, the number of studies using this method is astonishingly small. Although a 2D analysis system can only serve for gait patterns with strides close to the sagittal plane in the walking direction^17^, there are still various indications for its use in preventive and rehabilitative settings with a need for assessing kinematic and spatio-temporal parameters of level walking in the sagittal plane. The commonly used system TEMPLO™ which also provides optional integration of analog devices has no published data concerning the reliability and validity of the derived data. Therefore, this study was performed to assess (i) the validity of the video-based 2D system TEMPLO™ against a 3DGA reference standard, as well as (ii) the inter-tester reliability for three raters, and (iii) the test–retest for one rater, by means of 3DGA.

## Methods

### Study design

**Figure 1:**
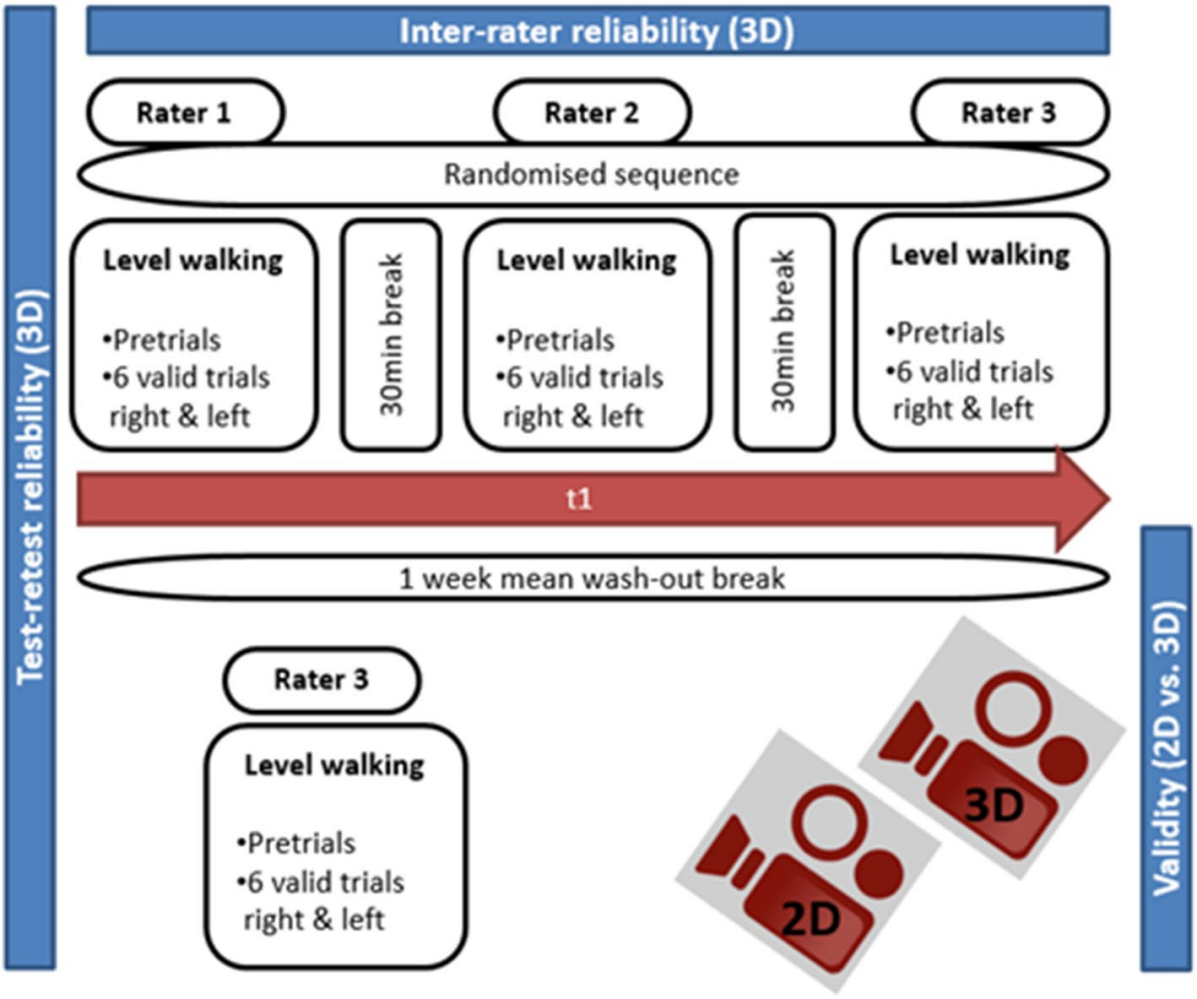
Flow diagram of the study design.

After giving informed consent, participants were examined for eligibility criteria. Gait examination started with three pre-trials and a calibration routine after the first rater had applied the marker set. Participants completed six valid trials for both, the right and the left side. Afterwards, markers were taken off. Before the next rater applied the marker set, a 30-min break was allowed in order to avoid skin irritation. The third rater finally repeated the procedure. To period effects, the order in which raters applied the marker set was randomized by a prepared random sequence list. A wash-out phase of mean 1-week was foreseen before the procedure was repeated by rater three only. All gait assessments were simultaneously captured by the 2D motion analysis system TEMPLO™ (Contemplas, Kempten, Germany) and the 3D motion analyses system VICON™ (Vicon Motion Systems Ltd, Oxford, United Kingdom) (Fig. 1). No changes were made to the trial design or eligibility criteria after the study has commenced.

### Participants

Potential participants were invited via the FH Campus Wien-University of Applied Sciences in-house info screens to be screened for eligibility. No monetary or other incentives were offered. Eligibility criteria were (i) age from 18 to 30 years, (ii) measured body mass index (BMI) from 18.5 to 24.99 kg/m^2^, and (iii) no musculoskeletal abnormalities in the lower extremity and/or spine. Data were collected in the movement lab of FH Campus Wien-University of Applied Sciences. Based on comparable publications, we hypothesized a sample of 22 subjects to be sufficiently powered for this study^18^. This would include a drop-out rate of 10%. Based on their profession, the three physiotherapists acting as raters in this study may be considered advanced in placing markers after a period of training sessions^9^, as opposed to non-experienced operators and examiners, not educated in anatomical palpation. Training sessions were supervised by rater three and an additional experienced gait analyst, acting in the role of the clinical trial monitor. The two training sessions consisted of marker placement, measuring anatomical distances, and participant instruction, which was performed by all three raters. In each assessment, the highly experienced monitor and the moderately experienced rater three checked and discussed the quality of the aspects mentioned.

All participants were enrolled by the principal investigator. Due to the nature of the assessments being studied, no arrangements were made related to allocation concealment or participant blinding. The ethical committee of the Medical University of Vienna approved the study protocol (1195/2016) and all participants provided written informed consent, before starting data collection. The Austrian Federal Office for Safety in Health Care (BASG) approved this clinical evaluation of a medical device (Ref-Nr 9119547) according to EN ISO 14155. The study was conducted in accordance with the approved study protocol, which is in accordance with the European Medicines Agency “ICH Good clinical practice” scientific guideline and the Declaration of Helsinki.

### Instrumentation

A 3D optoelectronic system controlled by the software Nexus 2.4 (Vicon Motion Systems Ltd., Oxford, United Kingdom) with reflective skin-markers of 14 mm diameter was used as reference standard. Fourteen cameras (200 Hz frame rate) were synchronized with two floor-mounted force plates (AMTI, Watertown, United States) recording at a 1000 Hz frame rate. Markers were placed according to the VICON Plug-in-Gait Lower Body model (PiG).

The 2D system was driven by the software TEMPLO™ (Contemplas, Kempten, Germany). Highspeed cameras (acA640gc-120, Basler AG, Ahrensburg, Germany) with a resolution of 480 × 640 pixels and a frame rate of 100 Hz captured videos for the right and left strides respectively. Cameras were connected to the computer via PoE-switch (GS108P, Netgear Inc., San José, CA, USA). Parallel to the camera view, spotlights illuminated the reflective markers for contrast reasons during data recording (Supplementary Figs.1, 4, 5). The camera images were calibrated using a one-meter by one-meter calibration object placed normal to the walkway (Supplementary Figs. 2, 3). Camera images captured a view of 3.36 m in length and 1.58 m in height from a distance of 2.5 m, which results in a resolution of 0.53 cm in length and 0.33 cm in height per pixel, which is sufficiently accurate with regard to measurement errors caused by soft tissue artefacts^19^. The positions of the cameras were optimized for capturing the strides with valid force plate strikes of the leg near to the relevant camera. For the segmental model (Supplementary Fig. 8), an additional marker was placed over the apex of the great trochanter, representing the hip joint. Due to central projection effects concerning metric calibration for 2D systems, identifying and data extraction for Heel Strike of the contralateral leg to the camera position was not possible.

### Procedure and data processing

In the clinical examination, the physiological range of motion of the spine and lower extremities were tested by an experienced physiotherapist to rule out any musculoskeletal abnormalities. BMI was calculated from measured body weight (M-420, Marsden Weighing Machine Group Ltd., Rotherham, United Kingdom) and height (Seca 213, Seca GmbH & Co KG, Hamburg, Germany). The length of the lower leg was measured from the floor to the medial knee joint space by one rater for all subjects. The following distances were measured by each rater and used for individual data processing: malleolus medialis to spina iliaca anterior superior, right to left spina iliaca anterior superior, and knee and ankle width.

After attaching the skin-markers, a static calibration trial was captured and joint centers were calculated. Participants were then asked to walk from a starting mark at self-selected speed with elbows bent for visibility of the pelvis and hip markers^20^. The recording trials started when they had become habituated to the arm constraint, their walking pattern appeared observationally stable, and the start mark had been optimized for valid force plate strikes. Six valid trials were recorded and averaged, with one left and one right step fully placed on the corresponding force plate.

Raw 3D data underwent the standard PiG pipeline using a 5th-order Woltering filter (mean square error-value: 20) of trajectories. Heel Strike and Toe-Off were determined using the standard event detection function with a 20 N force threshold. After time normalization in Polygon 4.2 (Vicon Motion Systems Ltd., Oxford, United Kingdom), data curves were exported to a spreadsheet for final parameter extraction.

For processing the predefined 2D gait parameters (as listed in Table 2), a segmental model, angle algorithms, and spatio-temporal parameter (STP) algorithms (Supplementary Fig. 9–16) were developed in Motus 10.1 (Contemplas, Kempten, Germany). Using this template, videos (Supplementary Fig. 6, 7) were imported from Templo™, and marker trajectories were automatically tracked. For detection of the event Heel Strike, customized algorithms using linear velocity and acceleration of the marker placed on the dorsal heel were used. Toe-Off was identified using parameters derived from the second toe marker coordinates. After running the above-mentioned calculations and applying a Butterworth 6 Hz 2nd-order filter^21^, data curves were pasted into ProEMG 2.1 (Prophysics AG, Kloten, Switzerland) for time normalization. Final parameter extraction was performed using the datasheet.

### Statistical analysis

Metric outcome parameters were first checked for outliers and normal distribution. Outliers were defined as an exclusion criterion if they deviated more than two standard deviations from the sample’s mean. Normal distribution was tested with the Shapiro–Wilk test and graphical inspections of Q–Q plots.

Agreement of the 2D with the 3D system (concurrent validity), as well as the test–retest reliability of rater three marker application consistency between two measurement days, was expressed by intraclass correlation coefficients (ICC, 3.k), i.e. absolute agreement, average measures, two-way mixed. Furthermore, mean differences were tested by repeated sample t-tests with Cohen´s d as standardized effect size and graphically visualized by Bland–Altman plots. Consistency between the three raters applying the marker set (interrater reliability) was expressed by ICC (2.k), i.e. absolute agreement, average measures, two-way random. Besides the ICCs, mean differences were tested by repeated sample ANOVA with eta-squared as standardized effect size.

For validity, ICC values above 0.7^22^ and for reliability, ICC values above 0.75^23^ were considered acceptable. A level of at least 0.9 was considered acceptable if the measure is used for decisions about an individual, rather than in a group of patients or a clinical trial^24^. The standard error of measurement (SEM = SD √ (1 − ICC)) was calculated as a further reliability metric, where the precision of the measurement is expressed in the unit of the specific outcome (e.g. °). In this context, the standard deviation for all test scores was derived from the total sum of squares of the ICC’s ANOVA (SD = √ (SS/ (n − 1))^25,26^. All statistical analyses were carried out with IBM SPSS statistics version 28 (IBM Corp., Armonk, NY). No additional subgroup or adjusted analysis was conducted. Alpha was set at 0.05. However, exact p-values have been reported.

## Results

### Sample characteristics

Age and anthropometric characteristics of the sample are summarized in Table 1. Twenty-one (21) subjects were screened for eligibility, and 19 thereof who passed the functional check were randomly assigned to a predefined sequence of three raters applying the marker set. All of these 19 participants completed all study procedures, including the retest where markers were applied by rater three only. One participant was excluded from further analysis due to outcome values differing more than two standard deviations from the sample’s mean^27^, resulting in a sample size of 18 participants (10 of which women). Technical issues occurred in terms of markers not being covered by the 2D camera view. Hence, outcomes related to the right ankle could not be analyzed in the 2D assessment for six participants (n = 12), and outcomes related to the right knee could not be analyzed in the 2D assessment for one participant (n = 17). This was because the right camera could not capture the individually placed first step due to its positioning. Follow-up assessments were conducted after a mean washout phase of six (min 1; max 18) days.

**Table 1:** Age and anthropometric characteristics, mean (standard deviation).

	n	Age (years)	Weight (kg)	Height (cm)	BMI (kg/m^2^)	vel-d1 (m/s)	vel-d2 (m/s)
Men	8	23.5 (2.7)	70.1 (4.6)	182.8 (4.5)	21.0 (1.7)	1.41 (0.08)	1.44 (0.13)
Women	10	21.9 (3.8)	59.7 (3.6)	168.3 (3.8)	21.1 (1.2)	1.42 (0.12)	1.45 (0.12)
All	18	22.6 (3.3)	64.3 (6.6)	174.7 (8.4)	21.1 (1.4)	1.42 (0.10)	1.45 (0.12)

*BMI* body mass index, based on measured data; *vel-d1* left leg walking speed derived from 3D-system at day 1; *vel-d2* left leg walking speed derived from 3D-system at day 2.

### Concurrent validity

We found acceptable (> 0.7) to excellent and statistically significant agreement of the 2D system with the 3D reference system in the assessed kinematic and spatio-temporal parameters, except for the parameters RoM hip and ankle with moderate ICC values, as well as toe-off and RoM pelvis with low ICC values. Absolute mean differences ranged from very small (e.g. 0.01 m/s velocity) to fairly high (e.g. 11.56° RoM pelvis). Due to the very low variability, several small differences were statistically significant (Table 2).

**Table 2:** Concurrent validity of selected outcomes assessed with TEMPLO (2D) against VICON (3D) as reference method, n = 18.

		Mean (SD)	ICC (CI_95_)	p^a^	p^b^	Δ^c^	d^d^
Left leg							
Stride time (s)	3D	1.02 (0.06)	0.97 (0.51–0.99)	< 0.01	< 0.01	0.02	1.2
	2D	1.00 (0.05)					
Toe-off (% gait cycle)	3D	59.0 (0.9)	0.26 (−0.15 to 0.65)	0.01	< 0.01	−2.01	−2.2
	2D	61.0 (1.0)					
Stride length (m)	3D	1.44 (0.07)	0.98 (0.88–0.99)	< 0.01	< 0.01	0.01	0.8
	2D	1.43 (0.06)					
Velocity (m/s)	3D	1.42 (0.10)	0.99 (0.98–0.99)	< 0.01	0.04	−0.01	−0.5
	2D	1.43 (0.10)					
RoM hip (°)	3D	44.1 (3.7)	0.45 (−0.92 to 0.82)	< 0.01	< 0.01	6.78	3.2
	2D	37.3 (3.4)					
RoM knee (°)^e^	3D	59.9 (4.5)	0.88 (0.68–0.96)	< 0.01	0.10	−1.18	−0.4
	2D	61.1 (4.7)					
RoM ankle (°)^f^	3D	35.5 (5.9)	0.41 (−0.05 to 0.80)	< 0.01	< 0.01	11.56	4.4
	2D	24.0 (4.4)					
RoM pelvis (°)	3D	3.3 (0.6)	0.18 (−0.18 to 0.47)	0.23	< 0.01	−2.48	−1.6
	2D	5.8 (1.6)					
Right leg							
Stride time (s)	3D	1.02 (0.05)	0.98 (0.57–0.99)	< 0.01	< 0.01	0.01	1.3
	2D	1.00 (0.05)					
Toe-off (% gait cycle)	3D	59.1 (0.9)	0.32 (−0.17 to 0.72)	0.01	< 0.01	−2.01	−2.2
	2D	61.2 (1.1)					
Stride length (m)	3D	1.42 (0.07)	0.97 (0.74–0.99)	< 0.01	< 0.01	0.02	1.0
	2D	1.40 (0.07)					
Velocity (m/s)	3D	1.40 (0.10)	0.99 (0.98–0.99)	< 0.01	0.77	< 0.01	< 0.1
	2D	1.40 (0.10)					
RoM hip (°)	3D	43.1 (3.7)	0.47 (-0.15—0.83)	< 0.01	< 0.01	5.99	2.4
	2D	37.2 (3.4)					
RoM knee (°)^e^	3D	60.0 (5.5)	0.86 (0.62–0.95)	< 0.01	0.52	−0.57	−0.2
	2D	60.6 (4.7)					
RoM ankle (°)^f^	3D	35.0 (7.4)	0.48 (−0.17 to 0.85)	< 0.01	< 0.01	10.37	2.3
	2D	24.7 (5.0)					
RoM pelvis (°)	3D	3.2 (0.5)	0.07 (−0.09 to 0.34)	0.16	< 0.01	−2.63	−2.7
	2D	5.8 (1.0)					

*Mean* (*SD*) mean (standard deviation), *ICC* intra class correlation coefficient (3.k): absolute agreement, average measures, two-way mixed with 95% confidence interval.

^a^p-value derived from ICC.

^b^p derived from t-test.

^c^Mean difference.

^d^Cohen’s d.

^e^n = 17.

^f^n = 12.

**Figure 2:**
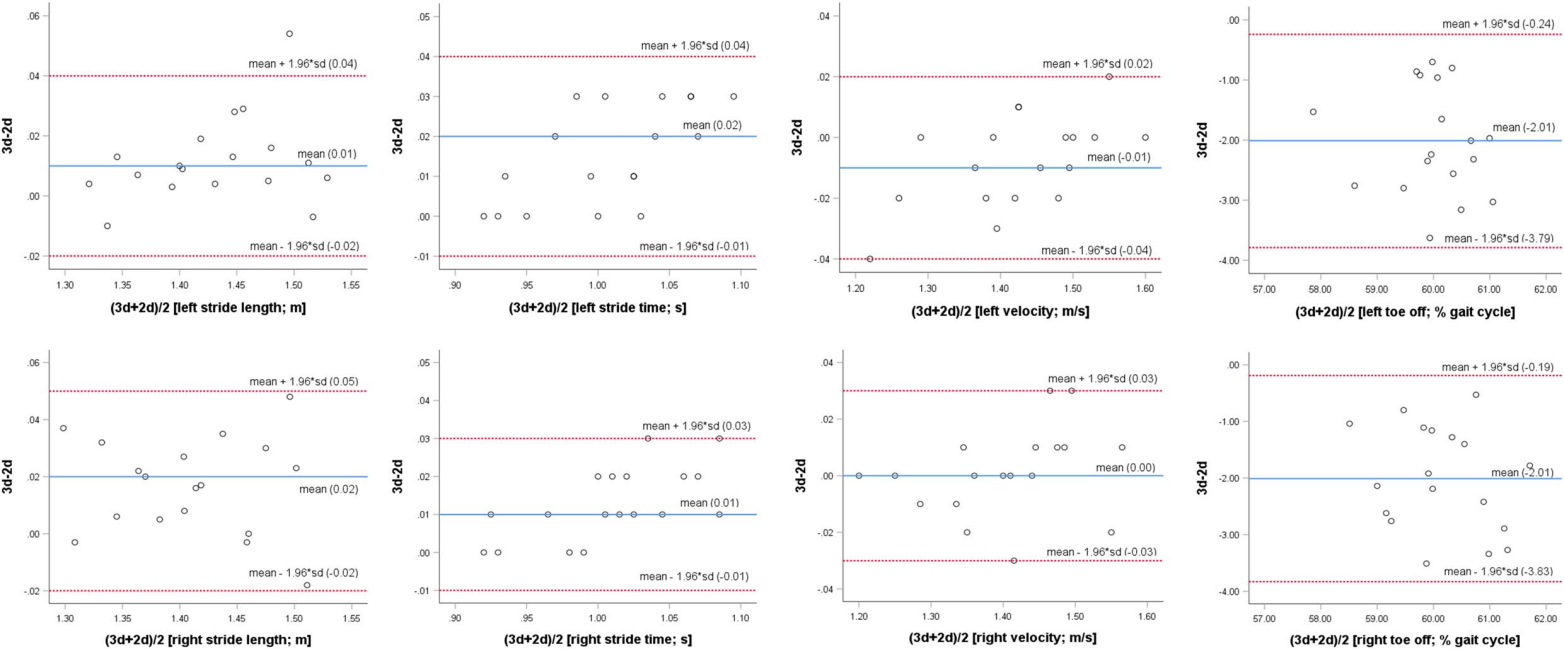
Bland–Altman plots for selected outcomes assessed with TEMPLO (2D) against VICON (3D) as reference method, n = 18; blue lines indicate mean differences and red lines 95% limits of agreement.

Bland–Altman plots show mean differences including their 95% limits of agreement for selected parameters (Fig. 2).

### Reliability

We found acceptable (ICC > 0.75) to excellent and statistically significant consistency between three raters for all spatio-temporal and kinematic parameters when assessed with 3DGA. Consistency that is also acceptable for decisions about an individual (> 0.9) was found for stride time, step length, step time, and RoM hip. For RoM parameters, standard error of measurement ranged from 0.23° to 2.86°. Significant differences across the three raters did not occur for any of the parameters (Table 3).

**Table 3:** Inter-rater reliability of selected outcomes assessed with VICON (3D). n = 18.

		Mean (SD)	CV (%)	ICC (CI_95_)	p^a^	SEM	p^b^	eta^2c^
Left leg								
Stride time (s)	Rater 1	1.02 (0.06)	6.0	0.97 (0.94–0.99)	< 0.01	0.01	0.999	0.000
	Rater 2	1.02 (0.06)	5.5					
	Rater 3	1.02 (0.06)	5.5					
Toe-off (% gait cycle)	Rater 1	59.3 (0.8)	1.3	0.89 (0.75–0.95)	< 0.01	0.27	0.496	0.027
	Rater 2	59.0 (0.8)	1.3					
	Rater 3	59.0 (0.9)	1.5					
Stride length (m)	Rater 1	1.42 (0.08)	5.5	0.95 (0.89–0.98)	< 0.01	0.01	0.680	0.015
	Rater 2	1.43 (0.07)	4.6					
	Rater 3	1.44 (0.07)	4.6					
Step length (m)	Rater 1	0.71 (0.04)	6.3	0.96 (0.92–0.99)	< 0.01	0.01	0.951	0.002
	Rater 2	0.72 (0.04)	5.4					
	Rater 3	0.71 (0.04)	5.3					
Step time (s)	Rater 1	0.51 (0.03)	6.0	0.98 (0.95–0.99)	< 0.01	0.00	0.987	0.001
	Rater 2	0.51 (0.03)	6.3					
	Rater 3	0.51 (0.03)	5.9					
RoM ankle (°)^d^	Rater 1	34.2 (5.8)	17.1	0.89 (0.73–0.96)	< 0.01	2.31	0.193	0.062
	Rater 2	38.4 (8.7)	22.7					
	Rater 3	35.5 (5.9)	16.7					
RoM knee (°)^e^	Rater 1	59.5 (5.7)	9.6	0.87 (0.71–0.95)	< 0.01	1.98	0.314	0.044
	Rater 2	57.3 (6.1)	10.6					
	Rater 3	59.9 (4.5)	7.6					
RoM hip (°)	Rater 1	45.0 (3.5)	7.7	0.95 (0.88—0.98)	< 0.01	0.83	0.533	0.024
	Rater 2	45.5 (4.3)	9.4					
	Rater 3	44.1 (3.7)	8.3					
RoM pelvis (°)	Rater 1	3.3 (0.6)	18.8	0.86 (0.69–0.94)	< 0.01	0.23	0.981	0.001
	Rater 2	3.3 (0.7)	22.3					
	Rater 3	3.3 (0.6)	17.0					
Right leg								
Stride time (s)	Rater 1	1.02 (0.06)	5.8	0.97 (0.98–0.99)	< 0.01	0.01	0.996	0.000
	Rater 2	1.02 (0.06)	5.5					
	Rater 3	1.02 (0.05)	5.4					
Toe-off (% gait cycle)	Rater 1	59.4 (0.9)	1.5	0.92 (0.83–0.97)	< 0.01	0.26	0.630	0.018
	Rater 2	59.4 (1.0)	1.6					
	Rater 3	59.1 (0.9)	1.5					
Stride length (m)	Rater 1	1.40 (0.08)	5.4	0.95 (0.89–0.98)	< 0.01	0.02	0.754	0.011
	Rater 2	1.41 (0.07)	4.7					
	Rater 3	1.42 (0.07)	4.6					
Step length (m)	Rater 1	0.69 (0.03)	4.8	0.90 (0.77–0.96)	< 0.01	0.01	0.369	0.038
	Rater 2	0.70 (0.03)	4.3					
	Rater 3	0.70 (0.03)	4.5					
Step time (s)	Rater 1	0.50 (0.03)	5.6	0.95 (0.89–0.98)	< 0.01	0.01	0.868	0.006
	Rater 2	0.50 (0.02)	4.6					
	Rater 3	0.50 (0.03)	5.3					
RoM ankle (°)^d^	Rater 1	34.2 (6.4)	18.7	0.86 (0.69–0.94)	< 0.01	2.86	0.393	0.036
	Rater 2	37.7 (9.8)	26.1					
	Rater 3	35.8 (6.3)	17.5					
RoM knee (°)^e^	Rater 1	59.7 (5.0)	8.4	0.89 (0.75–0.95)	< 0.01	1.68	0.959	0.002
	Rater 2	59.5 (4.9)	8.3					
	Rater 3	60.0 (5.3)	8.9					
RoM hip (°)	Rater 1	43.5 (3.2)	7.3	0.95 (0.88–0.98)	< 0.01	0.80	0.861	0.006
	Rater 2	43.8 (3.6)	8.3					
	Rater 3	43.1 (3.7)	8.5					
RoM pelvis (°)	Rater 1	3.2 (0.7)	21.8	0.87 (0.72–0.95)	< 0.01	0.23	0.975	0.001
	Rater 2	3.1 (0.7)	23.5					
	Rater 3	3.2 (0.5)	17.3					

*Mean* (*SD*) mean (standard deviation), *CV* coefficient of variation, *ICC* intra class correlation coefficient (2.k): absolute agreement, average measures, two-way random with 95% confidence interval, *SEM* standard error of measurement, derived from ICC.

^a^p-value derived from ICC.

^b^p-value derived from ANOVA.

^c^Effect size estimated by eta^2^.

^d^n = 12.

^e^n = 17.

We found acceptable (ICC > 0.75) to excellent and statistically significant consistency between test and retest of one rater for most of the parameters when assessed with 3DGA. Consistency that is also acceptable for decisions about an individual (> 0.9) was found for RoM hip, RoM knee (left leg only), RoM ankle, stride time, and step time (left leg only). Toe-off resulted in ICCs close to 0.75 threshold. Only values for RoM pelvis (left 0.22; right 0.53) were below the clinical acceptable threshold. For the RoM parameters, SEM ranged from 0.4° to 1.9°. Absolute mean differences ranged from very small (e.g. 0.01 s step time) to fairly small (e.g. 1.51° RoM knee) (Table 4). Mean walking velocity ranged from 1.40 to 1.42 m/s for the left leg, and 1.39 to 1.40 m/s for the right.

**Table 4:** Test re-test reliability of selected outcomes assessed with VICON (3D), n = 18.

		Mean (SD)	CV	ICC (CI_95_)	p^a^	SEM	p^b^	Δ^c^	d^d^
Left leg									
Stride time (s)	Test	1.02 (0.06)	5.5	0.90 (0.74–0.96)	< 0.01	0.02	0.15	0.01	0.4
	Re-test	1.00 (0.06)	6.3						
Toe-off (% gait cycle)	Test	59.0 (0.9)	1.5	0.73 (0.26–0.90)	0.01	0.45	0.98	< 0.01	< 0.1
	Re-test	59.0 (0.9)	1.5						
Stride length (m)	Test	1.44 (0.07)	4.6	0.86 (0.63–0.95)	< 0.01	0.03	0.40	0.01	−0.2
	Re-test	1.45 (0.07)	5.2						
Step length (m)	Test	0.71 (0.04)	5.3	0.87 (0.63–0.95)	< 0.01	0.02	0.07	−0.01	−0.5
	Re-test	0.73 (0.05)	6.6						
Step time (s)	Test	0.51 (0.03)	5.9	0.91 (0.76–0.97)	< 0.01	0.01	0.16	< 0.01	0.3
	Re-test	0.51 (0.04)	7.5						
RoM hip (°)	Test	44.1 (3.7)	8.3	0.94 (0.51–0.98)	< 0.01	0.95	0.01	−1.37	−1.0
	Re-test	45.6 (3.8)	8.3						
RoM knee (°)^e^	Test	59.9 (4.5)	7.6	0.93 (0.76–0.97)	< 0.01	1.23	0.03	−1.24	−0.6
	Re-test	61.2 (4.6)	7.5						
RoM ankle (°)^f^	Test	35.5 (35.5)	16.7	0.93 (0.82–0.98)	< 0.01	1.63	0.97	0.03	< 0.1
	Re-test	35.5 (35.5)	19.0						
RoM pelvis (°)	Test	3.3 (0.6)	17.0	0.22 (−0.87 to 0.69)	0.30	0.55	0.12	−0.31	−0.4
	Re-test	3.6 (0.6)	17.8						
Right leg									
Stride time (s)	Test	1.02 (0.05)	5.4	0.93 (0.74–0.98)	< 0.01	0.01	0.01	0.02	0.7
	Re-test	1.00 (0.06)	5.9						
Toe-off (% gait cycle)	Test	59.1 (0.9)	1.5	0.75 (0.33–0.91)	0.00	0.44	0.54	−0.12	−0.1
	Re-test	59.3 (0.9)	1.5						
Stride length (m)	Test	1.42 (0.06)	4.6	0.83 (0.57–0.94)	< 0.01	0.03	0.20	−0.02	−0.3
	Re-test	1.44 (0.08)	5.4						
Step length (m)	Test	0.70 (0.03)	4.5	0.71 (0.22–0.89)	0.01	0.02	0.59	< −0.01	−0.1
	Re-test	0.71 (0.03)	4.7						
Step time (s)	Test	0.50 (0.03)	5.3	0.89 (0.59–0.97)	< 0.01	0.01	0.01	0.01	0.7
	Re-test	0.50 (0.03)	5.3						
RoM hip (°)	Test	43.1 (3.7)	8.5	0.91 (0.67–0.97)	< 0.01	1.10	0.01	−1.19	−0.6
	Re-test	44.3 (3.4)	7.8						
RoM knee (°)^e^	Test	60.0 (5.3)	8.9	0.86 (0.62–0.95)	< 0.01	1.92	0.08	−1.51	−0.4
	Re-test	61.5 (4.9)	7.9						
RoM ankle (°)^f^	Test	35.8 (6.3)	17.5	0.92 (0.78–0.97)	< 0.01	1.76	0.06	1.51	0.5
	Re-test	34.3 (6.6)	19.1						
RoM pelvis (°)	Test	3.2 (0.5)	17.3	0.53 (−0.14 to 0.81)	0.05	0.43	0.06	−0.32	−0.5
	Re-test	3.5 (0.7)	19.3						

*Mean* (*SD*) mean (standard deviation), *CV* coefficient of variation (%), *ICC* intra class correlation coefficient (3.k): absolute agreement, average measures, two-way mixed with 95% confidence interval, *SEM *standard error of measurement derived from ICC.

^a^p-value derived from ICC.

^b^p derived from t-test.

^c^Δ mean difference.

^d^Cohen’s d.

^e^n = 17.

^f^n = 12.

## Discussion

This study provides deeper insights into the validity and reliability of a video-based system for assessing basic sagittal gait parameters. Considering the agreement of 2D derived values with the 3D reference system, both statistically significant and clinically relevant deviations were found for the parameters RoM hip and RoM ankle, where the 2D system generated lower RoM values.

Stride time, stride length, velocity, and RoM knee showed at least acceptable agreement and fairly small deviations of the 2D system with the 3D reference. Stride time, for instance, gave a mean system difference of 0.02 s, with limits of agreement between −0.01 and 0.04 s. For this and the other aforementioned parameters, the 2D system achieves an accuracy that is acceptable for many clinical applications. The low toe-off ICC value (left 0.26; right 0.32) might be due to the 2D-marker-based calculation versus the 3D method using force-plate thresholds. For RoM pelvis the agreement with the reference method was only weak, which might be caused by the varying view of the pelvic markers between 2D and 3D tracking. Although an acceptable agreement was achieved, the deviation from the reference method was fairly large for RoM hip and RoM ankle. The latter could be caused additionally by the camera setup, as the right ankle was captured farthest from the center of the camera view. Regarding RoM hip the differing view and following tracked coordinates of pelvic markers for the 2D and 3D technology might be of high relevance. To minimize lens distortion-related errors in 2D sagittal gait analysis, scan cameras with a resolution above two megapixels and a minimum distance between the sidewalk and the camera of 3.2 m can be recommended^5^. A possible variation of the distance from the camera to the sagittal plane of walking should not essentially bias the obtained results as participants were walking on a 0.5 m narrow walkway, which should facilitate a rather linear walking regarding the calibration plane.

One limitation is that the algorithms used for event detection were developed based on existing literature^28^ and observed agreement using the video. But spatio-temporal parameters mainly had an ICC close to one and a not clinically relevant standard error of measurement (derived from ICC). Through data fusion with spatio-temporal parameter-specific systems, this could even be optimized^29^. The levels of agreement found in this study when comparing 2D with 3D are somewhat worse when compared to the results of a study in which time parameters were validated for a markerless system^2^. For knee RoM Peebles et al. found an ICC of 0.94^30^, which is slightly higher than in our study (0.86–0.88). However, an absolute comparison between these studies is limited, as the participants in the Peebles study performed treadmill running and absolute values for angles were reported differently.

Inter-rater consistency resulted in ICCs ranging from 0.86 to 0.97 with no significant deviations across the three raters. Compared to a recent study conducted on a treadmill using a webcam recording at 30 Hz, ICC for repeatability was similar for hip and knee RoM^31^, supporting the findings of our study. Test–retest consistency was predominantly weaker than inter-rater consistency. Although the sample consisted of healthy, young, movement proficient participants, the wide range of 1 to 18 (mean 6) days between test and retest may have influenced intra-rater outcomes to some extent. However, there was no statistically significant correlation between the actual duration of wash-out phase and the ICC. In summary, most parameters achieved acceptable ICCs, and absolute mean differences were very small. Yet, ICCs considered acceptable for evaluating repeated scores of an individual (> 0.9) were not achieved for all parameters. For the interpretation of repeated assessments of a single client, clinicians are therefore advised to calculate the so-called minimum detectable change (MDC) based on the values of the standard error of measurement (derived from ICC) given in Table 4. MDC_95_ indicates the required change in a repeated measurement that would exceed the test–retest variability of the outcome with a 95% confidence level (MDC_95_ = SEM × 1.96 × √2)^32^.

It is assumed that both inter-rater and test–retest inconsistency result from a mixture of the variability of the analysis system itself, the rater-dependent marker application, and the patient’s gait pattern. Gait patterns may also be influenced by the patient's attempt to hit the force plates, which is recognizable to some extent in most laboratories and can be monitored to a limited extent visually or by assessing potential stride variability. The combination of these components reflects the actual real-life situation, but the extent to which each of these three components contributes to the inconsistency remains unclear and is subject for future research. Therefore, a design would need to be developed that compares identical movements to eliminate gait variability as an influencing factor. However, the higher test–retest inconsistency (compared to inter-rater inconsistency) observed in most cases is likely due to higher variability in the gait pattern between two measurement days. In our study, gait variability was minimized because participants were mainly exercise proficient and accommodation trials were performed on the walkway prior to gait assessment. Considering the influence of the technology used and the algorithms for processing parameters, a standardized data generation of gait data is urgently needed. Especially methods of kinematic assessments should be stated with a reference regarding the measurement properties. Additionally, a consensus group could provide a list of criterion-proven parameters with a related essential minimum standard of the instrumentation used. Based on such standards, marker based 2D-video gait analysis might be superior to the currently available markerless technologies in evaluation of gait in overweight and obese persons concerning sagittal hip and knee RoM.

## Conclusions

2D gait analysis provides a possibility to accurately assess parameters such as sagittal knee RoM, stride time, stride length, and gait velocity in a healthy population with a generally stable gait pattern. However, this may be different in specific patient populations. However, the parameters toe-off and pelvic RoM are not correctly captured by the 2D gait system and the accuracy of the other RoM parameters is limited and their applicability thus depends on the accuracy demands. Further, clinicians should keep in mind that 2D gait analysis provides relative angles and not joint-center-based calculation. Inter-rater and test–retest reliability of the 3DGA are generally acceptable, except for the parameter pelvic RoM. Nevertheless, the expertise of the raters in using the system should be taken into account when considering reliability and validity in interpreting findings. Clinical practitioners are advised to use the MDC to interpret whether a change detected in repeated measurements of a single client exceeds the test–retest variability of the outcome with a 95% confidence level.

### Supplementary Information

Supplementary Figures.

## Data Availability

Processed data will be made available upon reasonable request and for non-commercial purposes after publication of the results by contacting K.W.

## References

[1] McGinley JL, Baker R, Wolfe R, Morris ME (2009). The reliability of three-dimensional kinematic gait measurements: A systematic review. Gait Posture.

[2] Verlekar TT (2019). Estimation and validation of temporal gait features using a markerless 2D video system. Comput. Methods Programs Biomed..

[3] Ugbolue UC (2013). The evaluation of an inexpensive, 2D, video based gait assessment system for clinical use. Gait Posture.

[4] Rudisch J (2021). Agreement and consistency of five different clinical gait analysis systems in the assessment of spatiotemporal gait parameters. Gait Posture.

[5] Michelini A, Eshraghi A, Andrysek J (2020). Two-dimensional video gait analysis: A systematic review of reliability, validity, and best practice considerations. Prosthet. Orthot. Int..

[6] Eastlack ME, Arvidson J, Snyder-Mackler L, Danoff JV, McGarvey CL (1991). Interrater reliability of videotaped observational gait-analysis assessments. Phys. Ther..

[7] Kaufman K (2016). Reliability of 3D gait data across multiple laboratories. Gait Posture.

[8] Lee M, Song C, Lee K, Shin D, Shin S (2014). Agreement between the spatio-temporal gait parameters from treadmill-based photoelectric cell and the instrumented treadmill system in healthy young adults and stroke patients. Med. Sci. Monit..

[9] Leigh RJ, Pohl MB, Ferber R (2014). Does tester experience influence the reliability with which 3D gait kinematics are collected in healthy adults?. Phys. Ther. Sport.

[10] Cutlip RG, Mancinelli C, Huber F, DiPasquale J (2000). Evaluation of an instrumented walkway for measurement of the kinematic parameters of gait. Gait Posture.

[11] Reed LF, Urry SR, Wearing SC (2013). Reliability of spatiotemporal and kinetic gait parameters determined by a new instrumented treadmill system. BMC Musculoskelet. Disord..

[12] Lienhard K, Schneider D, Maffiuletti NA (2013). Validity of the Optogait photoelectric system for the assessment of spatiotemporal gait parameters. Med. Eng. Phys..

[13] Bessone V, Hoschele N, Schwirtz A, Seiberl W (2019). Validation of a new inertial measurement unit system based on different dynamic movements for future in-field applications. Sports Biomech..

[14] Mentiplay BF (2015). Gait assessment using the Microsoft Xbox One Kinect: Concurrent validity and inter-day reliability of spatiotemporal and kinematic variables. J. Biomech..

[15] Clark RA, Bower KJ, Mentiplay BF, Paterson K, Pua YH (2013). Concurrent validity of the Microsoft Kinect for assessment of spatiotemporal gait variables. J. Biomech..

[16] Parks MT, Wang Z, Siu KC (2019). Current low-cost video-based motion analysis options for clinical rehabilitation: A systematic review. Phys. Ther..

[17] Grunt S (2010). Reproducibility and validity of video screen measurements of gait in children with spastic cerebral palsy. Gait Posture.

[18] Walter SD, Eliasziw M, Donner A (1998). Sample size and optimal designs for reliability studies. Stat. Med..

[19] Leardini A, Chiari L, Della Croce U, Cappozzo A (2005). Human movement analysis using stereophotogrammetry. Part 3. Soft tissue artifact assessment and compensation. Gait Posture.

[20] Trehan SK, Wolff AL, Gibbons M, Hillstrom HJ, Daluiski A (2015). The effect of simulated elbow contracture on temporal and distance gait parameters. Gait Posture.

[21] Tully EA, Fotoohabadi MR, Galea MP (2005). Sagittal spine and lower limb movement during sit-to-stand in healthy young subjects. Gait Posture.

[22] Fitzpatrick R, Davey C, Buxton MJ, Jones DR (1998). Evaluating patient-based outcome measures for use in clinical trials. Health Technol. Assess..

[23] Heineman, A. *Rehabilitation Measures Database* http://www.rehabmeasures.org/rehabweb/rhstats.aspx (2010).

[24] Nunnally, J. C. & Bernstein, I. H. *Psychometric theory*. 3rd edn, 752 (McGraw-Hill, 1994).

[25] Weir JP (2005). Quantifying test–retest reliability using the intraclass correlation coefficient and the SEM. J. Strength Cond. Res..

[26] Koo TK, Li MY (2016). A guideline of selecting and reporting intraclass correlation coefficients for reliability research. J. Chiropr. Med..

[27] Sangeux M, Polak J (2015). A simple method to choose the most representative stride and detect outliers. Gait Posture.

[28] O'Connor CM, Thorpe SK, O'Malley MJ, Vaughan CL (2007). Automatic detection of gait events using kinematic data. Gait Posture.

[29] van Bloemendaal M (2019). Concurrent validity and reliability of a low-cost gait analysis system for assessment of spatiotemporal gait parameters. J. Rehabil. Med..

[30] Peebles AT, Carroll MM, Socha JJ, Schmitt D, Queen RM (2021). Validity of using automated two-dimensional video analysis to measure continuous sagittal plane running kinematics. Ann. Biomed. Eng..

[31] Saner RJ, Washabaugh EP, Krishnan C (2017). Reliable sagittal plane kinematic gait assessments are feasible using low-cost webcam technology. Gait Posture.

[32] Haley SM, Fragala-Pinkham MA (2006). Interpreting change scores of tests and measures used in physical therapy. Phys. Ther..

