# Supplementary material for: Validity of assessing level walking with the 2D motion analysis software TEMPLO and reliability of 3D marker application
Source: Sci Rep. 2024 Jan 16;14:1427. doi: 10.1038/s41598-024-52053-z (PMC10792076; doi:10.1038/s41598-024-52053-z)
Supplement: Supplementary file 1 — Supplementary Figures. [file 41598_2024_52053_MOESM1_ESM.pdf]

Supplementary material

„Validity of assessing level walking with a 2D motion analysis system and reliability of 3D marker application"

- **Illustration of the system setup**
  Relevant for this study are the cameras of the vicon system ( ), force plates (FP1, FP2) and the basler Aca640 cameras with attached spots (1a, 1b). Not relevant for this study are additionally measured walking speed (light gate) and the spatio-temporal parameter system OptoGait (blue bars).

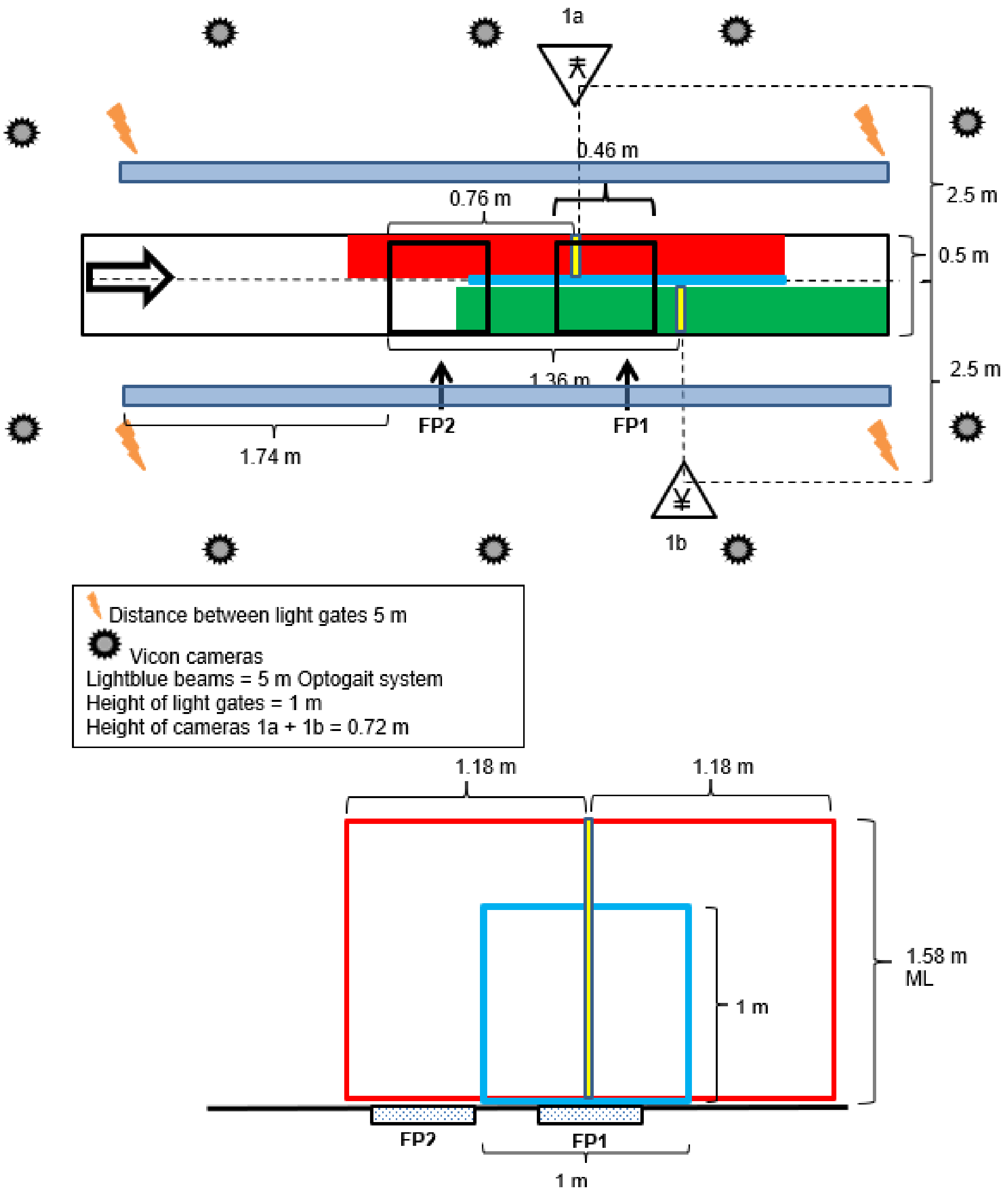

Figure 1: upper drawing shows the positions and distances of the force plates, 2D video cameras (1a, 1b) and the middle marks for the calibration object; lower drawing shows in blue the calibration object and red the achieved camera view.

- **Screenshots of the calibration video**, which was done on each assessment day. Video was imported to Vicon Motus software, tracked and attached as calibration file to the following processing of participants trials of the relevant day. Examples of a calibration video set is presented in figure 2 and 3.

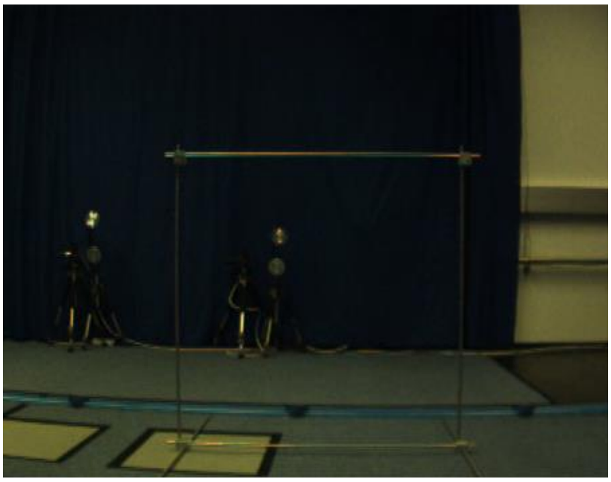

Figure 2: calibration video right camera

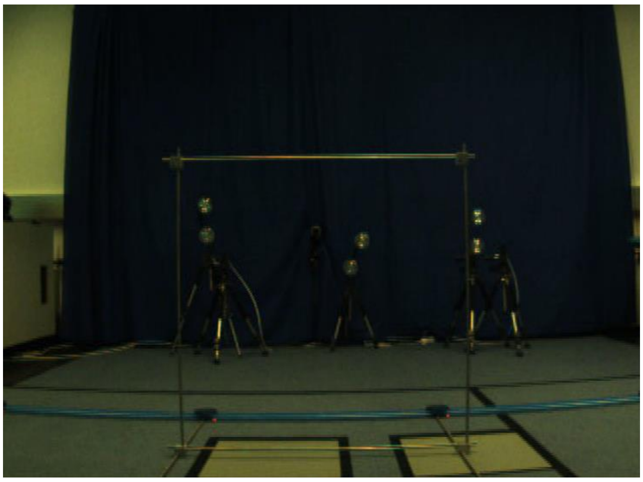

Figure 3: calibration video left camera

- **Views of the lab setting** are presented in figure 4 and 5.
  - For the final assessments the walkway in the middle was covered with the blue carpet to minimise spot light reflexions.
  - The second camera set was not for the gait assessment reported in this study.

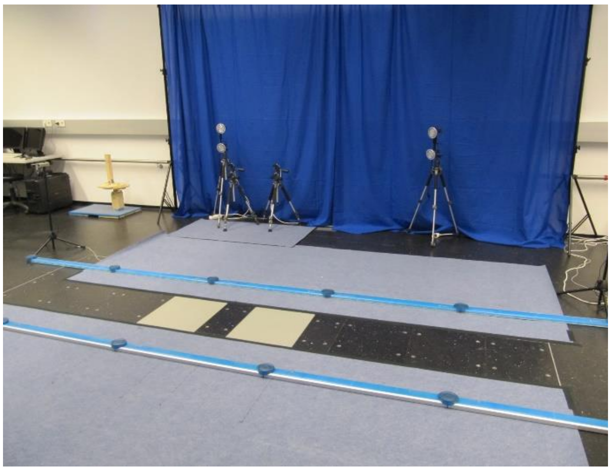

Figure 4: system setting in the lab

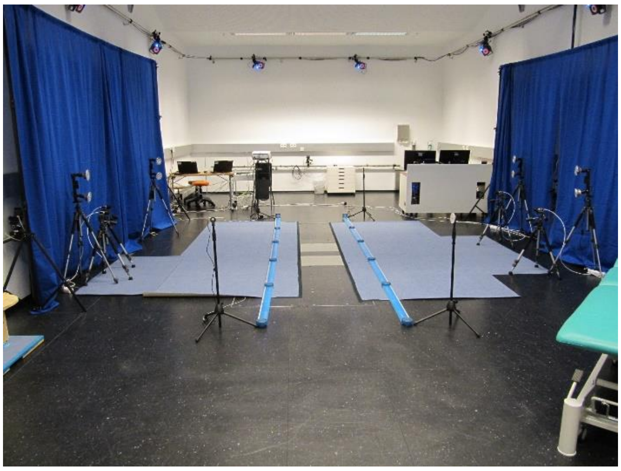

Figure 5: overview system setting in the lab

- **Screenshots of one recorded walking trial** for the right (figure 6) and left (figure 7) side. (Note: screenshots are not synchronised)

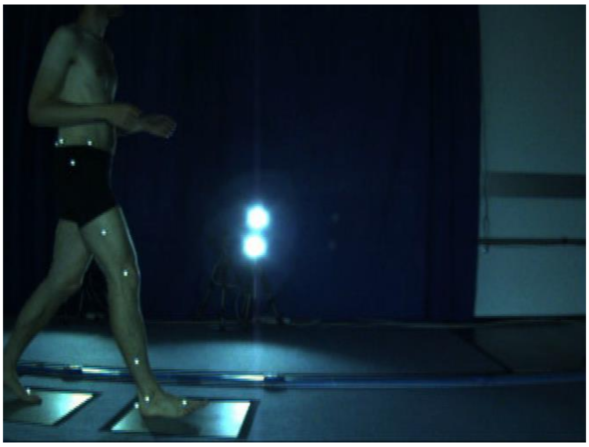

Figure 6: example single walking trial right leg

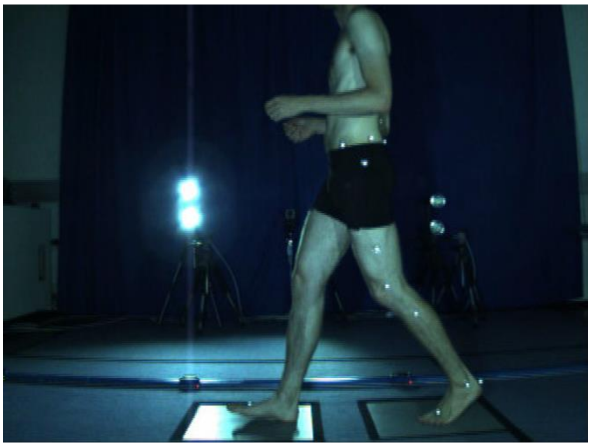

Figure 7: example single walking trial left leg

- **Markermodel and joint angles are illustrated in figure 8.**
  - Markers additional to the PiG-Markerset were the LPEL (left pelvis half) and LHIP (left apex greater trochanter)

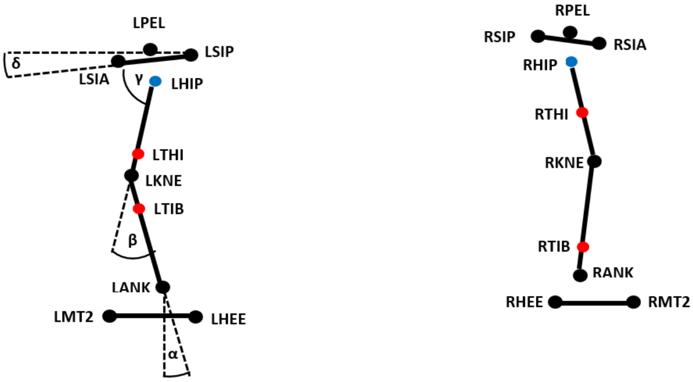

Figure 8: segmental model with angles

  - Definition of segments

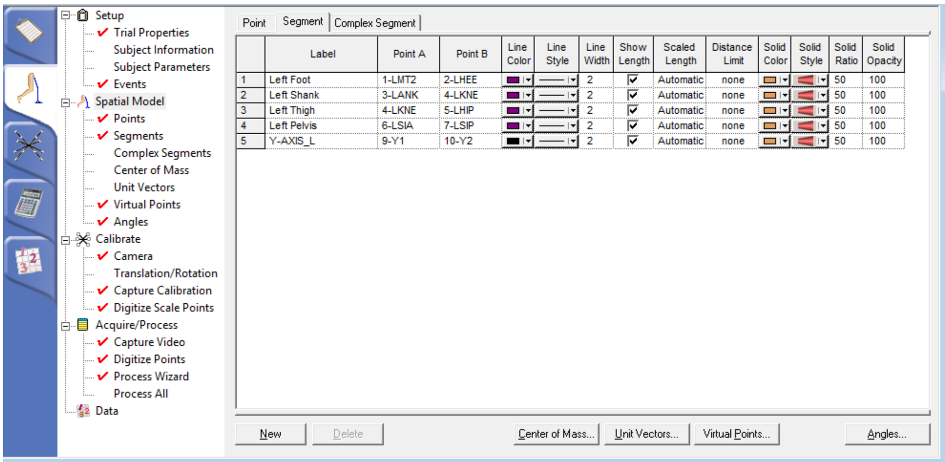

Figure 9: Definition of segments

- Pelvic tilt was calculated between horizontal plane and the LSIA-LSIP segment

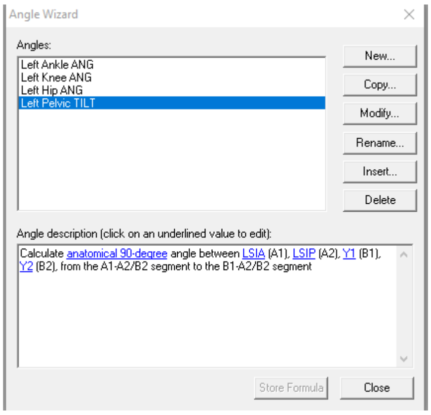

Figure 10: Calculation pelvic tilt angle

- Hip sagittal angle was calculated between the LSIA-LSIP and LHIP-LKNE segment

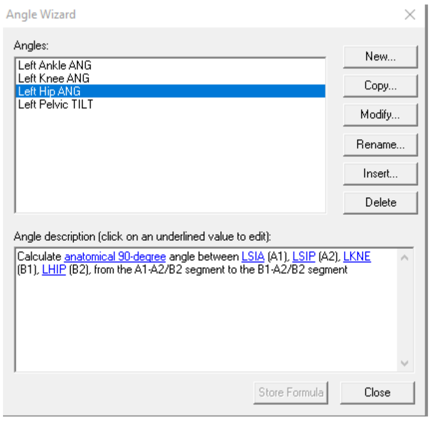

Figure 11: Calculation hip angle

- Knee sagittal angle was calculated between the LHIP-LKNE and LKNE-LANK segment

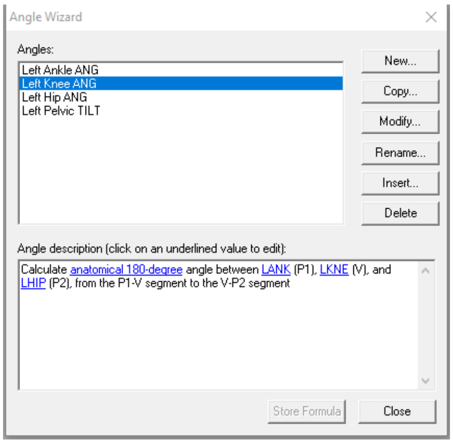

Figure 12: Calculation knee angle

- Ankle sagittal angle was calculated between the LKNE-LANK segment and the LMT2-LHEE segment

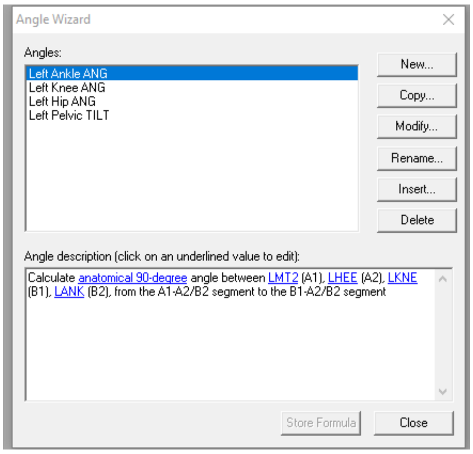

Figure 13: Calculation ankle angle

- **Event and parameter calculations** (performed in Vicon Motus Kinecalc)
  - StrideLength {Displacement(2DScaledCoordinates.LHEE.X,@IC1 First.0, 2DScaledCoordinates.LHEE.X,@IC2 First.0)}
  - StrideTime {INTERVALTIME(2DScaledCoordinates.LHEE.X,@IC1 First.0, 2DScaledCoordinates.LHEE.X,@IC2 First.0)}
  - StanceTime {INTERVALTIME(2DScaledCoordinates.LHEE.X,@IC1 First.0, 2DScaledCoordinates.LHEE.X,@TO First.0) /StrideTime }
  - SwingTime {INTERVALTIME(2DScaledCoordinates.LHEE.X,@TO First.0, 2DScaledCoordinates.LHEE.X,@IC2 First.0) /StrideTime}
  - Speed {StrideLength/StrideTime}
  - Initial Contact 1

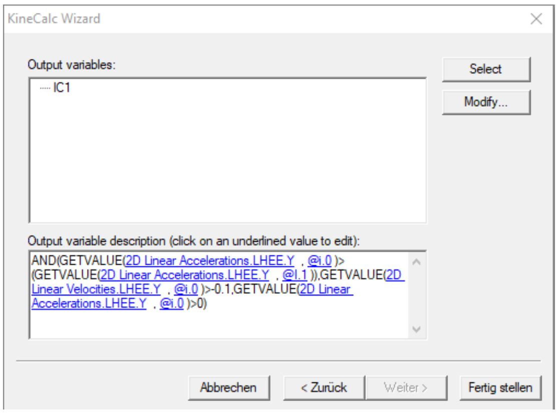

- **Figure 14:** Calculation initial contact 1

- Toe-Off

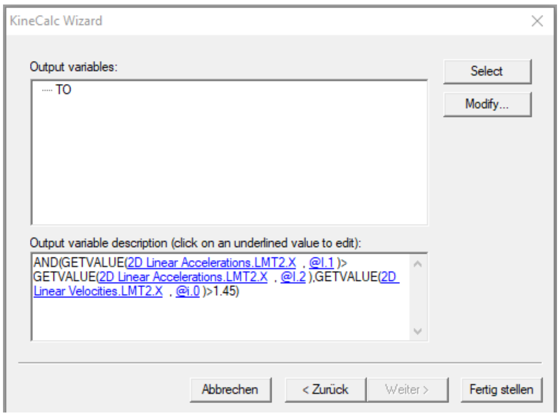

Figure 15: Calculation toe-off

- Initial Contact 2

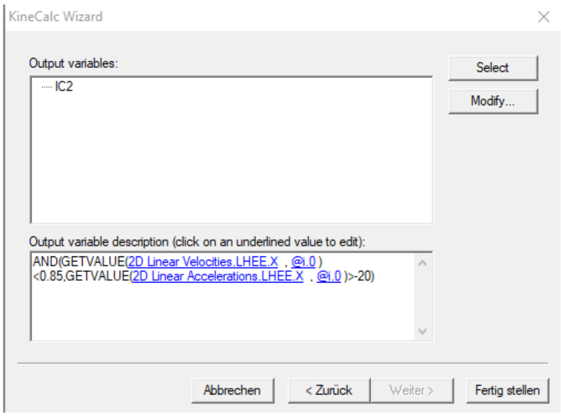

Figure 16: Calculation initial contact 2
